# Nicotinamide Mononucleotide Administration Triggers Macrophages Reprogramming and Alleviates Inflammation During Sepsis Induced by Experimental Peritonitis

**DOI:** 10.3389/fmolb.2022.895028

**Published:** 2022-06-27

**Authors:** Cécile Cros, Marielle Margier, Hélène Cannelle, Julie Charmetant, Nicolas Hulo, Laurent Laganier, Alessia Grozio, Matthias Canault

**Affiliations:** ^1^ Nuvamid SA, Lausanne, Switzerland; ^2^ LGD SAS, Velaux, France

**Keywords:** sepsis, peritonitis, nicotinamide mononucleotide, nicotinamide adenine dinucleotide, monocyte, macrophage

## Abstract

Peritonitis and subsequent sepsis lead to high morbidity and mortality in response to uncontrolled systemic inflammation primarily mediated by macrophages. Nicotinamide adenine dinucleotide (NAD+) is an important regulator of oxidative stress and immunoinflammatory responses. However, the effects of NAD+ replenishment during inflammatory activation are still poorly defined. Hence, we investigated whether the administration of β-nicotinamide mononucleotide (β-NMN), a natural biosynthetic precursor of NAD+, could modulate the macrophage phenotype and thereby ameliorate the dysregulated inflammatory response during sepsis. For this purpose, C57BL6 mice were subjected to the cecal ligation and puncture (CLP) model to provoke sepsis or were injected with thioglycolate to induce sterile peritonitis with recruitment and differentiation of macrophages into the inflamed peritoneal cavity. β-NMN was administered for 4 days after CLP and for 3 days post thioglycolate treatment where peritoneal macrophages were subsequently analyzed. In the CLP model, administration of β-NMN decreased bacterial load in blood and reduced clinical signs of distress and mortality during sepsis. These results were supported by transcriptomic analysis of hearts and lungs 24 h post CLP-induction, which revealed that β-NMN downregulated genes controlling the immuno-inflammatory response and upregulated genes involved in bioenergetic metabolism, mitochondria, and autophagy. In the thioglycolate model, a significant increase in the proportion of CD206 macrophages, marker of anti-inflammatory M2 phenotype, was detected on peritoneal exudate macrophages from β-NMN-administered mice. Transcriptomic signature of these macrophages after bacterial stimulation confirmed that β-NMN administration limited the pro-inflammatory M1 phenotype and induced the expression of specific markers of M2 type macrophages. Furthermore, our data show that β-NMN treatment significantly impacts NAD + metabolism. This shift in the macrophage phenotype and metabolism was accompanied by a reduction in phagolysosome acidification and secretion of inflammatory mediators in macrophages from β-NMN-treated mice suggesting a reduced pro-inflammatory activation. In conclusion, administration of β-NMN prevented clinical deterioration and improved survival during sepsis. These effects relied on shifts in the metabolism of organs that face up an increased energy requirement caused by bacterial infection and in innate immunity response, including reprogramming of macrophages from a highly inflammatory phenotype to an anti-inflammatory/pro-resolving profile.

## 1 Introduction

The activation of innate and adaptive immune responses against infection is crucial for the clinical evolution of peritonitis and sepsis. These conditions are characterized by a massive release of pro-inflammatory mediators that lead to uncontrolled inflammation, vascular injury and disseminated intravascular coagulation, and ultimately multiple organ failure and death (Jarczak et al., 2021). The innate immune response to infection during peritonitis and sepsis sequentially involves neutrophils and macrophages. While neutrophils are the first defensive line against pathogens, macrophages play a central role in initiating, maintaining, and resolving inflammation through the expression of numerous cytokines and growth factors (Hwang et al., 2019; Wang et al., 2020; Jarczak et al., 2021; Liu et al., 2021).

Depending on specific environmental stimuli, macrophages can differentiate into a pro-inflammatory or an anti-inflammation/pro-resolving phenotype, named M1 and M2 macrophages, respectively (Hwang et al., 2019; Wang et al., 2020; Liu et al., 2021). Tissue levels of M1 macrophages rise significantly in the early stage of infection as their primary function is to phagocyte and kill bacteria (Wang et al., 2020) as well as secrete pro-inflammatory cytokines (e.g., IL-1β, IL-6, and TNF-α) (Hwang et al., 2019; Wang et al., 2020; Liu et al., 2021). M2 macrophages appear at later stages of the infection to control and resolve inflammation and repair tissues and are characterized by their ability to secrete transforming growth factor β (TGF-β), Arg-1, CD206, and IL-10 (Wang et al., 2020). Accordingly, the subtle equilibrium between the M1 and M2 phenotype is responsible for efficient toxin clearance while ensuring proper tissue repair (Hwang et al., 2019). However, it is important to keep in mind that such an M1/M2 classification is mostly an *in vitro* paradigm that does not necessarily reflect the macrophage heterogeneity observed *in vivo* (Bonnardel and Guilliams, 2018; Guilliams et al., 2018).

In recent years, it has been shown that the polarization of macrophages toward the M1 or M2 phenotypes is also characterized by different metabolic signatures. In M1 macrophages, a “break” in the tricarboxylic acid (TCA) cycle and a shift toward glycolysis was demonstrated, whereas M2 macrophages favor oxidative phosphorylation (OXPHOS). Indeed, lipopolysaccharide (LPS), a known inducer of septic shock, downregulates mitochondrial respiration and reduces mitochondrial DNA (mtDNA) cellular content and ATP production while increasing glycolysis (Dang and Leelahavanichkul, 2020).

Nicotinamide adenine dinucleotide (NAD+) is a redox coenzyme and a key cofactor for NAD+-dependent enzymes including sirtuins, poly-ADP-ribose polymerases (PARPs), and CD38/157 ectoenzymes, involved in cellular metabolism, stress resistance, DNA repair and longevity (Garrido and Djouder, 2017). Mounting evidence suggests that NAD+ levels decline with age at a systemic level and associates its depletion with several hallmarks of aging and pathophysiological conditions (Verdin, 2015; Yoshino et al., 2018; Lautrup et al., 2019). Therefore, augmentation of NAD+ levels with various forms of biosynthetic precursors is emerging as a potential strategy to counteract a wide range of age-associated pathophysiological conditions such as cancer, diabetes, neurodegeneration, and inflammation (Covarrubias et al., 2021).

In mammalian cells, NAD+ synthesis primarily relies on the salvage pathway, in which nicotinamide (NAM) is recycled into β-nicotinamide mononucleotide (β-NMN), via nicotinamide phosphoribosyl transferase (NAMPT), and β-NMN is directly converted into NAD+ by nicotinamide mononucleotide adenylyl transferases (NMNATs) (Verdin, 2015). Interestingly, NAD+ is decreased during sepsis (Liaudet et al., 2001; Kwon et al., 2011) suggesting that boosting NAD+ levels could potentially ameliorate the clinical evolution of peritonitis and sepsis.

Indeed, it was demonstrated that macrophages mostly rely on the NAM-salvage pathway to sustain intracellular NAD+ levels. In particular, M2 macrophage polarization requires higher NAD+ levels, and β-NMN administration was able to rescue the oxidative phosphorylation reduced by FK866, a selective NAMPT inhibitor, in M2 macrophages (Covarrubias et al., 2020).

The potential role of NAD+ and its precursors in the peritonitis- and sepsis-associated inflammation was recently proposed. β-NMN treatment was shown to suppress the pro-inflammatory cytokine production in LPS-induced inflammation by decreasing cyclooxygenase-2 (COX-2) expression and inhibiting prostaglandin E2 (PGE2) production in macrophages (Liu et al., 2021). Lastly, another precursor of the salvage NAD+ biosynthesis, nicotinamide riboside (NR), was shown to reduce oxidative stress, inflammation, and caspase-3 activity in heart and lung tissues in a mouse model of sepsis induced by feces-injection in the peritoneum (FIP) (Hong et al., 2018). Here, we demonstrate that β-NMN administration significantly improved the clinical deterioration and the survival in septic mice likely by attenuating the dysregulated inflammation through the reprogramming of macrophages toward an anti-inflammatory/pro-resolving phenotype.

## 2 Materials and Methods

### 2.1 Animals

For this study, 7- to 12-week-old male C57BL/6 mice were obtained from the Janvier Labs (Le-Genest-Saint Isle, France). The mice were group-housed in the barrier facility of Ambiotis SAS (Toulouse, France) with 12/12 h light/dark cycles and received a regular chow diet *ad libitum*. Experimental procedures were reviewed and approved by the Animal Care and Ethics Committee (Ethics Committee of the US 006/CREFRE; reference number: APAFIS#22923-2019112512267652). All animal experiments were conducted by Ambiotis SAS.

### 2.2 Nicotinamide Mononucleotide Preparation

The β-NMN powder was obtained from Seneque Distribution (St Priest, France) and was dissolved in saline (NaCl 0.9%, vehicle). The β-NMN solution was prepared fresh before each administration and provided by intraperitoneal (i.p.) injection at 185 mg/kg (mouse body weight). The β-NMN regimen injected into mice was chosen based on allometric extrapolation from a human dose of 900 mg/day for a 60-kg individual, which is within the range of doses repeatedly administered to humans without generating observable adverse effects (Liao et al., 2021). Thus, with a conversion factor of 12.3 between mice and humans (Nair and Jacob, 2016), 900 mg for a 60-kg individual corresponds to 900/60 × 12.3 = 184.5 mg/kg.

### 2.3 Cecal Ligation and Puncture Model

Experimental peritonitis was induced by cecal ligation and punction (CLP) as previously described (Hubbard et al., 2005). Briefly, a laparotomy was realized on anesthetized mice with 3% isoflurane (ISO-VET^®^ 100%, Osalia, Paris, France), to exteriorize the cecum. For the induction of peritonitis and mid-grade sepsis, the cecum was ligated between its base and distal pole and perforated twice with a 23-gauge needle allowing the release of fecal material from both the mesenteric and anti-mesenteric penetration holes to ensure potency. The intestine was relocated on the abdominal cavity and the peritoneum, fasciae, abdominal musculature, and skin were closed by applying simple sutures. Animals were resuscitated through subcutaneous injection of prewarmed saline (37°C; 5 ml per 100 g body weight). A sham group of control mice also underwent a laparotomy procedure without cecal ligation and puncture. Administration of β-NMN (185 mg/kg/day) and vehicle (saline, 0.9% NaCl) were performed immediately after surgery, followed by daily injections for up to 4 days before euthanasia and the last treatment was 24 h before sacrifices. In these experiments, the animals were randomly divided in the three groups (*n* = 10 for the sham group, *n* = 6 for the vehicle group, and *n* = 6 for the β-NMN group) for cohort 1 and 2 groups (*n* = 17 for the vehicle group and *n* = 17 for the β-NMN group) for cohort 2. For cohort 1, 24 h after surgery, blood was collected in EDTA tubes through the retro-orbital sinus and used for 1) NAD+ and 1-methyl-nicotinamide (1-MeNAM) dosages, 2) cytokine level quantifications, and 3) immunophenotyping of circulating cells. Mice were then euthanized, and tissues (heart and lung) were harvested and used for 1) NAD+ and 1-MeNAM dosages, 2) histology analysis, and 3) RNA-seq transcriptome profiling.

In vehicle- and β-NMN-treated mice of cohort 2, the survival rate was recorded over a 4-day period, every 12 h during the first 2 days after surgery, and then once per day until the euthanasia. Rectal temperature was monitored immediately after the surgery/resuscitation procedure every 2–4 h for 24 h. Clinical scores were measured once daily for 4 days. Bacterial load was measured in blood from animals of cohort 2 (vehicle and β-NMN groups), 24 h after surgery/resuscitation. The clinical score was adapted according to the murine sepsis score (MSS) (Shrum et al., 2014; Mai et al., 2018).

### 2.4 Thioglycolate-Induced Peritonitis Model

To induce sterile peritonitis, mice were injected i. p. with 1 ml of Brewer thioglycolate medium (TGC, 4% in distilled water; Sigma-Aldrich, Munich, Germany). Mice were then randomly assigned to β-NMN (*n* = 6) and vehicle (*n* = 6) groups and were injected i.p. once daily for 3 days, with β-NMN (185 mg/kg/day) or saline (0.9% NaCl, vehicle), respectively. The first dose was co-injected with TGC and the last one 24 h before euthanasia. On day 3 post TGC injection, mice were anesthetized (3% Isoflurane) and blood was collected in EDTA tubes through the retro-orbital sinus for NAD+ and 1-MeNAM level quantifications. Mice were then euthanized, and peritoneal lavage was performed with 2 ml of phosphate-buffered saline (PBS). Peritoneal exudates were then centrifuged (450 g for 5 min at room temperature), and supernatants were stored and used for the analysis of NAD+ and 1-MeNAM levels. Pelleted cells were either used in flow cytometry to determine their immunophenotyping or cultured and stimulated with *Escherichia coli* (*E. coli*) to 1) determine their phagocytosis ability, 2) assess their viability and cytokine secretions, and 3) determine their transcriptome profiling.

### 2.5 Quantification of Plasma Cytokine and Chemokine Levels

Thirty-two different cytokines and chemokines were quantified simultaneously in plasma derived from mice in cohort 1 of the CLP model using the Mouse Cytokine/Chemokine Array 32-plex from Eve Technologies Corp (Calgary, Canada) using the Bio-PlexTM 200 system (Bio-Rad Laboratories, Hercules, CA, United States ), and a Milliplex Mouse Cytokine/Chemokine kit (Millipore, St. Charles, MO, United States ) according to the manufacturer’s protocols.

### 2.6 Bacterial Load Analysis

Bacterial DNA was extracted from the blood sample derived from mice in cohort 1 of the CLP model using the Quick-DNA Micropep Kit (Zymo Research, Irvine, CA, United States) following the manufacturer’s instructions. The microbial load was then quantified by qPCR targeting the bacterial 16S rRNA gene using an Applied Biosystems™ StepOnePlus™ system (Thermo Fisher Scientific, Waltham, MA, United States ) using the 16S RNA specific primer pair Bac-16S-FW (TTA-AAC-TCA-AAG-GAA-TTG-ACG-G) and Bac-16S-RV (CTC-ACG-RCA-CGA-GCT-GAC-GAC) (Eurogentec, Liege, Belgium). Gene copy number was calculated automatically based on the standard curve of the primer system using Applied Biosystems™ StepOnePlus™ Software.

### 2.7 Histology Analysis

Histology analysis was performed at Sciempath Labo (Larcay, France). Heart and lung tissues derived from mice in cohort 1 of the CLP model were fixed in ethanol and paraffin-embedded according to Sciempath Labo technical procedures. For each organ, 4–5 μm thick sections were obtained using a Microtome Tissue Tek^®^ Accu-Cut^®^ SRM 200 (Sakura Finetek, Torrance, CA, United States ) then stained with hematoxylin and eosin (H&E). The complete digitization of the H&E-stained tissue sections was performed using a Hamamatsu Nanozoomer at ×20 magnification. Each section was examined by a trained histopathologist from Sciempath Labo in a double-blinded way.

### 2.8 Peritoneal Macrophage Phenotyping

Macrophages from peritoneal exudate derived from mice in the TGC model (0.5 × 10^6^ cells) were resuspended in FACS buffer (PBS supplemented with 0.5% bovine serum albumin and 0.1% NaN_3_) and were labeled with the antibody mixes from panel 1 (CD45-APC; F4/80-Viogreen; MHCII-Vioblue; LFA-1-PeVio770; CD64-PE; CD16/32-Viobright FITC) (Miltenyi Biotec, Bergisch Gladbach, Germany) and panel 2 (CD45-APC; F4/80-Viogreen; CD64-PE; MHCII-Vioblue; CD38-PeVio770; CD206-FITC) (Miltenyi Biotec, Bergisch Gladbach, Germany) for 20 min at 4°C. Cells were then washed with FACS buffer and the relative fluorescence intensities were determined using a MACSQuant Vyb cytometer equipped with FlowLogic software (Miltenyi Biotec, Bergisch Gladbach, Germany).

### 2.9 Peritoneal Macrophage Stimulation With *E. Coli*

#### 2.9.1 Phagocytosis Assay

Pelleted cells from peritoneal lavages derived from mice in the TGC model were plated (1 × 10^6^ cells) in Hanks’ balanced salt solution with calcium and magnesium (HBSS^+/+^) for 2 h at 37 °C, 5% CO_2_. Nonadherent cells were discarded, and adherent macrophages were cultured in the presence of *E. coli* (15 × 10^6^ bacteria; MOI of 1:15) previously labeled with pHrodo Red succinimidyl ester according to the manufacturer’s recommendations (Thermo Fisher Scientific, Waltham, MA, United States). Since the pHRodo dye fluoresces only when in the acidic environment of phagolysosomes, it allows the specific detection of engulfed bacteria. Phagocytosis ability of peritoneal macrophages was monitored at 30 min, 2-, and 4-h by flow cytometry (MACSQuant Vyb cytometer equipped with FlowLogic software).

#### 2.9.2 Cytokine Secretion and Cell Viability

Pelleted cells from peritoneal lavages derived from mice in the TGC model were seeded in 96-well plates (0.15 × 10^6^ cells/well) in HBSS^+/+^ and stimulated with 2.5 × 10^6^ *E. coli* XL 10 Gold (Agilent Technologies, Santa Clara, CA, United States) for 24 h, at 37 °C, 5% CO_2_. After 6 h, the HBSS^+/+^ medium was supplemented with 10% fetal bovine serum and 1% penicillin–streptomycin–neomycin. At 24 h, supernatants were collected, and concentrations of cytokines (IL-6, IL-10, IL-12p70, and TNF-α) were determined by flow cytometry (MACSQuant Vyb cytometer equipped with FlowLogic software) using a bead-based immunoassay (AimPlex Mouse 4-Plex Custom assay kit; CliniSciences, Nanterre, France) following the manufacturer’s instructions. The cellular viability was assessed by adding AlamarBlue^®^ (Invitrogen, Carlsbad, CA, United States ) directly to cells in a culture medium (1:10; v:v) for 2 h. The conversion of resazurin to resorufin resulting in a pronounced color change was measured as an indicator of cell death. The absorbance (600 nm) was measured using the Infinite^®^ F500 spectrometer (Tecan, Männedorf, Switzerland).

### 2.10 RNA-Seq Analysis

#### 2.10.1 Sample Preparation and Sequencing

In the CLP model, tissues (10 mg) derived from mice of cohort 1 were treated with 400 µL RNAlater™ solution (Thermo Fisher Scientific, Waltham, MA, United States). In the TGC model, cells from peritoneal lavage were plated at 0.5 × 10^6^ cells/well in HBSS^+/+^ and stimulated for 6 h at 37 °C, 5% CO_2_, with *E. coli* (7.5 × 10^6^ bacteria; MOI of 1:15). Supernatants were discarded and cells were treated with 100 µl of RNAlater™ solution. All samples were submitted to Genewiz (Leipzig, Germany). Sequencing was performed on an Illumina NovaSeq™ 6,000 platform after total RNA extraction, poly(A) selection, and library generation. Single-end 150 bp sequencing reads without adaptors were analyzed.

#### 2.10.2 RNA-Seq Data Analysis

Approximately 30 million paired-end reads of 150 bp per sample were obtained and the quality of reads was checked with the fastqc tool. Sequences were mapped against the Mouse Reference Genome (assembly Mm10, UCSC, Dec. 2011) with the STAR program (version 2.7.0) (Dobin et al., 2013), and count tables containing the number of mapped reads per gene were produced with featureCounts (version 2.0.0). Count tables were then imported into R to do the differential expression analysis with edgeR (version 3.32.1) (Robinson et al., 2010). Library sizes were adjusted with a scaling factor calculated using a trimmed mean of M-values (TMM) between each pair of samples. The common dispersion and tagwise dispersions were estimated with the estimateDisp function. After negative binomial glm fitting the quasi-likelihood (QL), F-test was applied for the testing procedure. The order differentially expressed gene lists obtained with this procedure were then used to do gene set enrichment analysis (GSEA) on the Reactome pathway database with clusterProfiler functions (version 3.18.1) (Wu et al., 2021).

#### 2.10.3 RNA-Seq Data Visualization

Genes of interest were visualized with heatmap either generated with ggplot2 (version 3.3.5) or with ComplexHeatmap (version 2.6.2) when genes and samples were clustered. Pathways significantly enriched (q-value<0.02) were visualized with ggplot2 scripts. Redundant pathways and pathways irrelevant to a given tissue were removed.

### 2.11 Ultra-High Performance Liquid Chromatography Analysis

NAD+ and 1-MeNAM levels were measured from 1) blood (from cohort 1 of the CLP model and TGC model), 2) peritoneal fluid (from the TGC model), and 3) heart and lung from mice in cohort 1 of the CLP model. Samples were preliminarily acidified at a ratio of 1:4 (v:v; 0.5 N perchloric acid) for blood or peritoneal fluid and 10 mg of tissues in 400 µl perchloric acid (0.1 N). The latest was crushed prior to further analysis, using a Precellys Evolution Homogenizer equipped with a Cryolys Evolution cooling module (Bertin Instruments, Rockville, MD, United States).

All samples were then centrifugated at 12,000 g for 15 min. One hundred microliters of supernatant were added to 100 µl of 0.01% Difluoroacetic Acid (DFA) and the mixture was filtered at 0.22 μm on a MultiScreen HTS GV Filter Plate (EMD Millipore, Billerica, MA, United States). A volume of 5 µl was used for Ultra-High-Performance Liquid Chromatography (UHPLC) analysis. NAD+ and 1-MeNAM were separated using an Acquity HSS T3 column (15 cm × 2.2 mm x 1.8 μm) kept at a constant temperature (30°C). Mobil phase A consisted of 0.01% DFA and mobile phase B was 100% acetonitrile. The following linear gradient was run for 11 min at a flow rate of 0.45 ml/min: 0–1 min 100% A, 2.5 min 97.5% A, 7 min 5% A, 8.1 min 100% B. Standard curves were prepared in perchloric acid and samples were kept at 5°C in the autosampler tray prior injection. The Waters^®^ Acquity UPLC system was composed of a quaternary Solvent Manager (H-Class), FTN Sample Manager, and PDA eλ detector. NAD+ and 1-MeNAM were measured at 266 nm using the photodiode array detector and identified by retention time coincident with authentic standard (1.04 min for 1-MeNAM and 3.6 min for NAD+). Compound quantification was performed using Empower3^®^ software comparing peak area with external calibration curves. For the tissues, quantification was normalized to protein contents in homogenates using the Pierce BCA protein assay kit (Thermo Fisher Scientific, Waltham, MA, United States).

### 2.12 Statistical Analysis

Data shown are presented as mean values ± standard error of the mean. Outlier identification and statistical analyses were performed using GraphPad Prism software, version 9.3.0 (GraphPad Software Inc., San Diego, CA, United States). Differences between more than two groups of unpaired data were tested by parametric one-way ANOVA followed by Dunnett’s *post hoc* test, two-way ANOVA with Tukey correction, or nonparametric Kruskal–Wallis test followed by Dunnett’s *post hoc* test. Differences between only two groups of unpaired data were tested by the parametric *t*-test or the nonparametric Mann–Whitney test. The Mantel–Cox log rank test was performed to compare the survival of the different groups. Values of *p* < 0.05 were considered significant. *p < 0.05, **p < 0.01, ***p < 0.001, ****p < 0.0001.

## 3 Results

### 3.1 Treatment With β-NMN Prevents Mortality and Alleviates Clinical Manifestations During Sepsis

**FIGURE 1:**
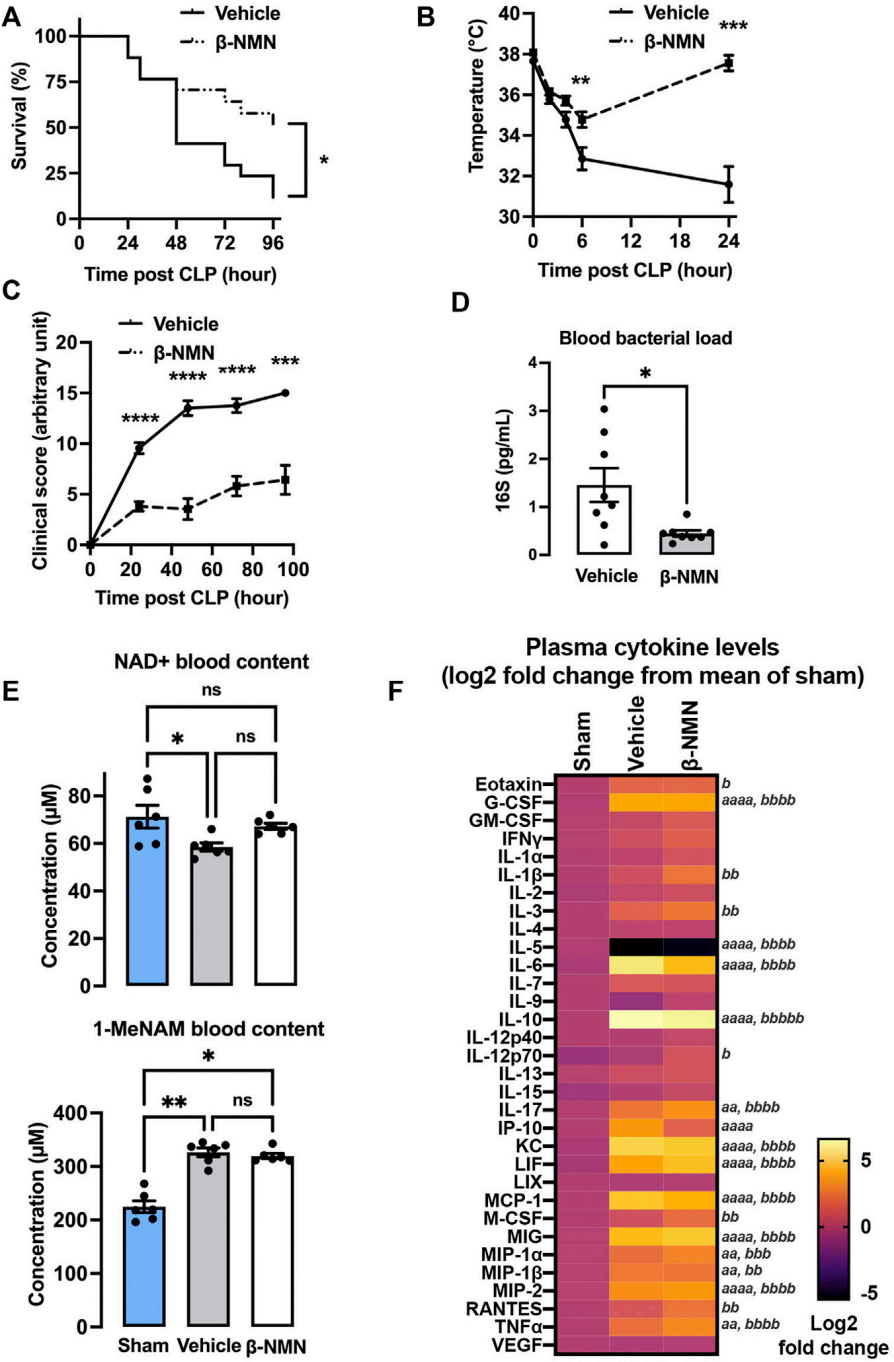
Survival, clinical, and biological parameters in the CLP-induced septic mouse model. Saline (0.9% NaCl; vehicle) or β-NMN at 185 mg/kg was immediately administered after CLP and every day for 4 days. **(A)** Survival was recorded every 12 h for the first 2 days and then once per day. Data are representative of two independent experiments and are mean ± SEM; n = 17 for each group; *p < 0.05 vs. vehicle. **(B)** Rectal temperature was monitored every 2-4 h for 1 day. Data are representative of two independent experiments and are mean ± SEM; n = 16 for each group; **p < 0.01; ***p < 0.001 vs. vehicle. **(C)** Clinical score was monitored once per day for 4 days and adapted according to the MSS score. Data are representative of two independent experiments and are mean ± SEM; n = 12–17 for each group; ***p < 0.001; ****p < 0.0001 vs. vehicle. **(D)** Blood of vehicle and β-NMN mice were collected 24 h post CLP, and bacterial DNA (16S) was extracted and quantified by qPCR. Data are mean ± SEM; n = 8 for each group; *p < 0.05 vs. vehicle. **(E)** Blood from sham, vehicle, and β-NMN mice were collected and acidified 24 h post CLP and NAD+, and 1-MeNAM were dosed by UHPLC. Data are means ± SEM; n = 6 for each group; ns = non-significant; *p < 0.05; **p < 0.001versus vehicle. **(F)** Heatmap of 32 inflammatory mediators. Plasma from the sham, vehicle, and β-NMN mice was collected 24 h post CLP, and 32 inflammatory mediators (cytokines, chemokines, and growth factors) were quantified using a Mouse Cytokine/Chemokine Array 32-plex assay. Data were transformed on Log 2-fold-change from the mean relative to the sham group and were displayed as a heatmap. n = 4-6 for each group; *a p* < 0.05; *aa p* < 0.01; *aaaa p* < 0.0001 vehicle vs. sham; *b p* < 0.05; *bb p* < 0.01; *bbb p* < 0.001; *bbbb p* < 0.0001 β-NMN vs. sham. Note: there is no significant difference between vehicle and β-NMN groups. G-CSF = granulocyte-colony stimulating factor; GM-CSF = granulocyte-macrophage colony-stimulating factor; IFN-γ = interferon gamma; IL = interleukin; IP-10 = interferon gamma-induced protein 10; KC = keratinocyte-derived chemokine; LIF = leukemia inhibitory factor; LIX = lipopolysaccharide-induced CXC chemokine; MCP = monocyte chemoattractant protein; M-CSF = macrophage colony-stimulating factor; MIG = monokine-induced by gamma interferon; MIP = macrophage inflammatory protein; RANTES = regulated on activation normal T cell expressed and secreted; TNF-α = tumor necrosis factor alpha; VEGF = vascular endothelial growth factor.

The CLP-induced peritonitis and septic shock model was specifically calibrated to reach 50% mortality between 36 and 48 h post induction in vehicle-treated animals. Survival was compared between β-NMN- and vehicle-treated animals over 4 days. A log-rank test analysis of the survival curves showed that the survival rate of the β-NMN-treated mice was significantly higher than that of the vehicle-treated group (Figure 1A). The median survival time calculated for the vehicle-treated group was 48 h with a survival rate of 12% after 4 days. On the other hand, survival time cannot be defined for the β-NMN-treated animals as their survival rate of 51% at day 4 post-CLP does not allow its determination (data not shown).

As expected, a significant decrease in body temperature was noticed in vehicle-treated mice as early as 2 h after surgery and peaked 24 h after CLP with a maximum drop of -6.07 ± 0.85 °C as compared to the basal presurgery temperature. β-NMN treatment alleviated temperature loss 6 h post-surgery and 24 h after induction of CLP. At 24 hours, the body temperature of β-NMN treated mice returned to the basal level, before surgery, and was significantly different compared to vehicle group (Figure 1B). Clinical parameters were then evaluated using the MSS score which is the average of scores given between 0 (normal) and 3 (severe changes from normal) for seven components including animal appearance, level of consciousness, spontaneous activity, response to stimulus, spontaneous eye-opening, and respiration quality. The clinical scores of β-NMN-treated animals remained significantly lower than those measured in the vehicle group all over the post-surgery observation period (Figure 1C).

### 3.2 β-NMN Treatment Decreases Bacterial Loads and Affects Plasma Cytokine Levels 24 h Post-CLP

Bacterial loads were evaluated in circulating blood, 24 h post-induction using bacterial DNA quantification by qPCR (Figure 1D). Blood bacterial loads were significantly lower in β-NMN- than vehicle-treated animals. In addition, β-NMN at 2 different concentrations (10 and 100 µM), only moderately reduced *in vitro* growth of 2 distinct bacterial strains (i.e., *E. coli* DH5α and methicillin-resistant *S. Aureus*) (Supplementary Figure S1), suggesting that the beneficial effects on survival and clinical parameters of β-NMN treatment are likely due to the stimulation of the host response to infection whereas the direct effect on bacterial growth. However, we did not notice any major differences in blood leukocyte counts (i.e., neutrophils, monocytes, B and T lymphocytes, NK cells, and dendritic cells) between β-NMN-treated and vehicle animals, 24 h after CLP (Supplementary Figure S2).

Circulating NAD+ levels were significantly reduced 24 h after induction of CLP and this significance is lost after administration of β-NMN. Interestingly, levels of circulating 1-MeNAM were significantly increased in the blood of animals that have undergone CLP regardless of the treatment they received (Figure 1E), suggesting that one administration of β-NMN was able to partially sustain the intracellular NAD+ level during the increased NAD+ -turnover induced by CLP.

The concentrations of 32 inflammatory mediators (cytokines, chemokines, and growth factors) were analyzed in the plasma of sham, vehicle- and β-NMN-treated mice, 24 h post-CLP. CLP induced significant increases or trends toward increases in the secretion of 13 inflammatory mediators (i.e., G-CSF, IL-6, IL-10, IL-17, CXCL-10 (IP-10), KC, LIF, MCP-1, MIG, MIP1-α and -β, MIP2, and TNF-α), whereas IL-5 levels were markedly decreased in mice subjected to CLP (vehicle and β-NMN vs. sham). Remarkably, even if not statistically significant, CXCL10 (IP-10) and IL-6 levels tend to decrease upon β-NMN treatment as compared to vehicle-treated animals (Figure 1F)

### 3.3 β-NMN Treatment Affects Early Transcriptomic Signatures in Heart and Lungs From Mice Subjected to CLP

**FIGURE 2:**
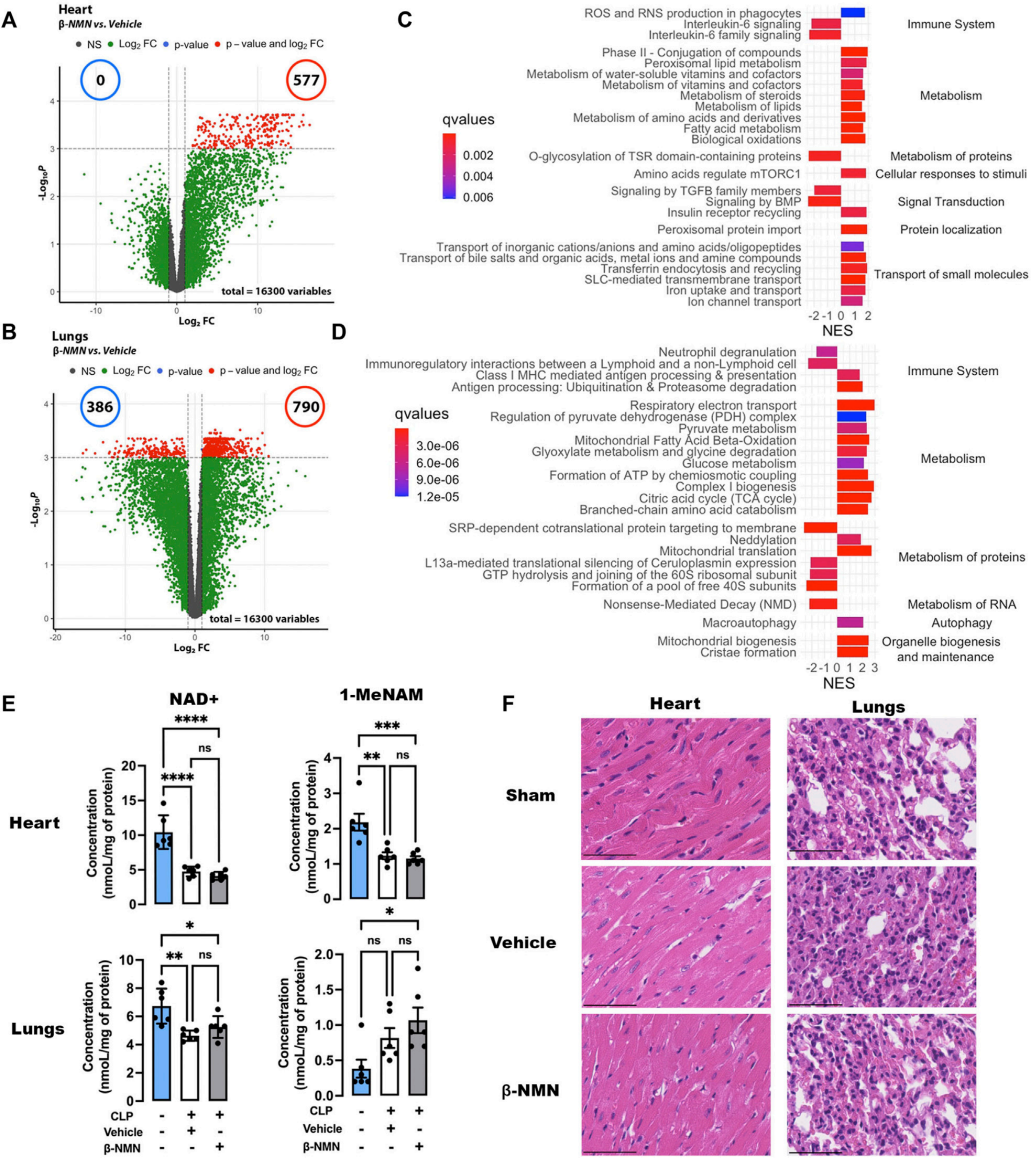
Transcriptomic signatures, NAD+ and 1-MeNAM contents, and histology analysis of the heart and lungs of mice subjected to CLP. Hearts and lungs from vehicle and β-NMN mice were collected 24 h post CLP. **(A,B)** Volcano plots of the differentially expressed genes in the hearts **(A)** and lungs **(B)**. Volcano plots display log2-transformed fold-changes (*x*-axis) vs. -log10 *p*-value (*y*-axis). The red dots represent the gene having fold-changes>2.0 and adjusted *p*-values<0.05 between the two groups. *n* = 3 for each group. **(C,D)** Enrichment plot of differentially expressed Reactome pathways in the hearts **(C)** and lungs **(D)**. Normalized enrichment score (NES) reflects the up- (NES>0) and down- (NES<0)expressed pathways, and gradient color reflects the adjusted *p*-values<0.01; *n* = 3 for each group. **(E)** NAD+ and 1-MeNAM contents of hearts and lungs. Data are means ± SEM; *n* = 6 for each group; ns = non-significant; *p < 0.05; **p < 0.01; ***p < 0.001; ****p < 0.0001. **(F)** Representative H&E section (x20) of the hearts and lungs from the sham, vehicle**,** and β-NMN mice. There was no histopathological finding in any of the heart and lung sections examined. n = 3 for each group.

Sepsis induces multi-organ failure leading to patients’ death, which is caused by the dysregulated host response to infection (Singer et al., 2016). We therefore investigated whether the beneficial effects of β-NMN treatment on mortality and clinical signs associated with septic shock might be due, at least in part, to the protective effects of β-NMN on vital organs. Thus, we performed gene expression profiling in the hearts and lungs collected 24 h after polymicrobial sepsis induction, in vehicle and β-NMN groups of animals (Figure 2). These organs were chosen as they are more frequently affected by sepsis, and their degree of dysfunction correlates with patients’ fate (Caraballo and Jaimes, 2019).

Pairwise comparison between mice treated with vehicle or β-NMN identified 1,157, and 1,968 deferentially expressed genes (adjusted *p*-value<0.05) in heart and lung samples, respectively, 24 h after CLP induction (Figures 2A,B).

Then, we performed gene set enrichment analysis (GSEA) using the Reactome database. This allowed us to highlight changes and alterations specific to one organ, but also those common to both organs upon treatment with β-NMN during the initial phase of the development of polymicrobial sepsis. Focusing on the top 24 impacted pathways, the analysis yielded 19 and 17 upregulated and 5 and 7 downregulated pathways in the heart and lungs, respectively (Figure 2C and 2D), the list of all impacted pathways can be consulted in the Supplementary Table S1. A common downregulation of pathways associated with the immune system emerges from the analysis of both organs, specifically, 2 out of 4 and 2 out of 3 pathways were downregulated in lungs and hearts, respectively. In hearts, β-NMN treatment decreases the expression of IL-6-related signaling. In lungs, we found that β-NMN downregulated innate immune responses through pathways affecting neutrophil activation (neutrophil degranulation), and genes involved in the communication between innate and acquired systems (i.e., immunoregulatory interactions between a lymphoid and a nonlymphoid cell). Interestingly, pathways of antigen presenting through the MHCI class receptor (Class I MHC mediated antigen processing and presentation), and antigen processing are both upregulated (Antigen processing: Ubiquitination and Proteasome degradation)

Common features of both organs also include upregulation of pathways of cell metabolism (upregulated/total: 9/9 and 10/10 pathways in lungs and hearts, respectively). Indeed, the current definition of sepsis implies not only alterations in the mechanisms of inflammation and immune responses but also dysfunction in the metabolism of all macronutrients—carbohydrates, lipids, and proteins (Wasyluk and Zwolak, 2021).

In the lungs, the treatment with β-NMN positively impacted carbohydrates metabolism with up-regulation of the pathways involved in glucose metabolism, and the TCA cycle.

We observed that β-NMN treatment also led to an upregulation of pathways involved in lipid metabolism. In particular, the pathways regulating fatty acid metabolism are activated in both organs and a major impact of β-NMN on general lipid metabolism was noted in hearts (i.e., biological oxidations, fatty acid metabolism, metabolism of lipids, steroids, and peroxisomal lipids).

In septic conditions, the early inflammatory phase is accompanied by an increased breakdown of protein stores (Michie, 1996), and this is paired with the shutdown of protein translation in organs (Hato et al., 2019). β-NMN administration appeared to increase the expression of genes involved in amino acid metabolism and catabolism in the heart and lungs (O-glycosylation of TSR domain-containing proteins, metabolism of amino acids and derivates, in the heart and branched chain amino acids catabolism in the lungs). However, in the lungs, a more complex regulation of protein metabolism was noted as we found an increase in the mitochondrial protein translation pathway but accompanied by a decrease in the expression of genes involved in translation initiation.

Sepsis-related energy production and metabolic disorders are thought to be caused by mitochondrial function alterations. Interestingly, β-NMN seems to have a positive effect on mitochondrial functions, markedly in the lungs where we observe an up-regulation of genes involved in mitochondrial dynamics, and biogenesis, which are essential mechanisms for maintaining mitochondria’s ability to slow sepsis damages.

Additionally, certain pathways are specifically altered in each organ. In hearts, β-NMN causes upregulation of pathways of cellular response to stimuli, transport of small molecule and protein localization (upregulated/total: 1/1, 6/6, and 1/1 pathways, respectively), and downregulated pathways associated with signal transduction (downregulated/total: 3/4 pathways). In the lungs, pathways of RNA metabolism are downregulated (downregulated/total: 1/1 pathway), whereas autophagy and organelle biogenesis are upregulated (upregulated/total: 1/1 and 2/2 pathways, respectively), upon β-NMN treatment.

NAD+ and 1-MeNAM levels in the heart and NAD+ in the lungs were significantly reduced after CLP induction. Administration of β-NMN did not prevent these decreases. In contrast, 1-MeNAM levels in the lungs tended to increase after CLP induction. β-NMN treatment enhanced this increase to statistical significance (Figure 2E).

In parallel, the histological analysis failed to show obvious pathological changes and dysfunctions induced by CLP neither in vehicle-treated nor in β-NMN-treated animals as compared to sham-operated animals (Figure 2F), highlighting the early effects of β-NMN on gene expression profiles in these organs even before visible alterations in tissue organization and function.

Our data support a significant effect of a single administration of β-NMN on the early phases of the development of sepsis and organ alterations.

### 3.4 The Presence of β-NMN During the Development of Sterile Peritonitis Alters the Phenotype of Resident and Recruited Cells in the Peritoneum

**FIGURE 3:**
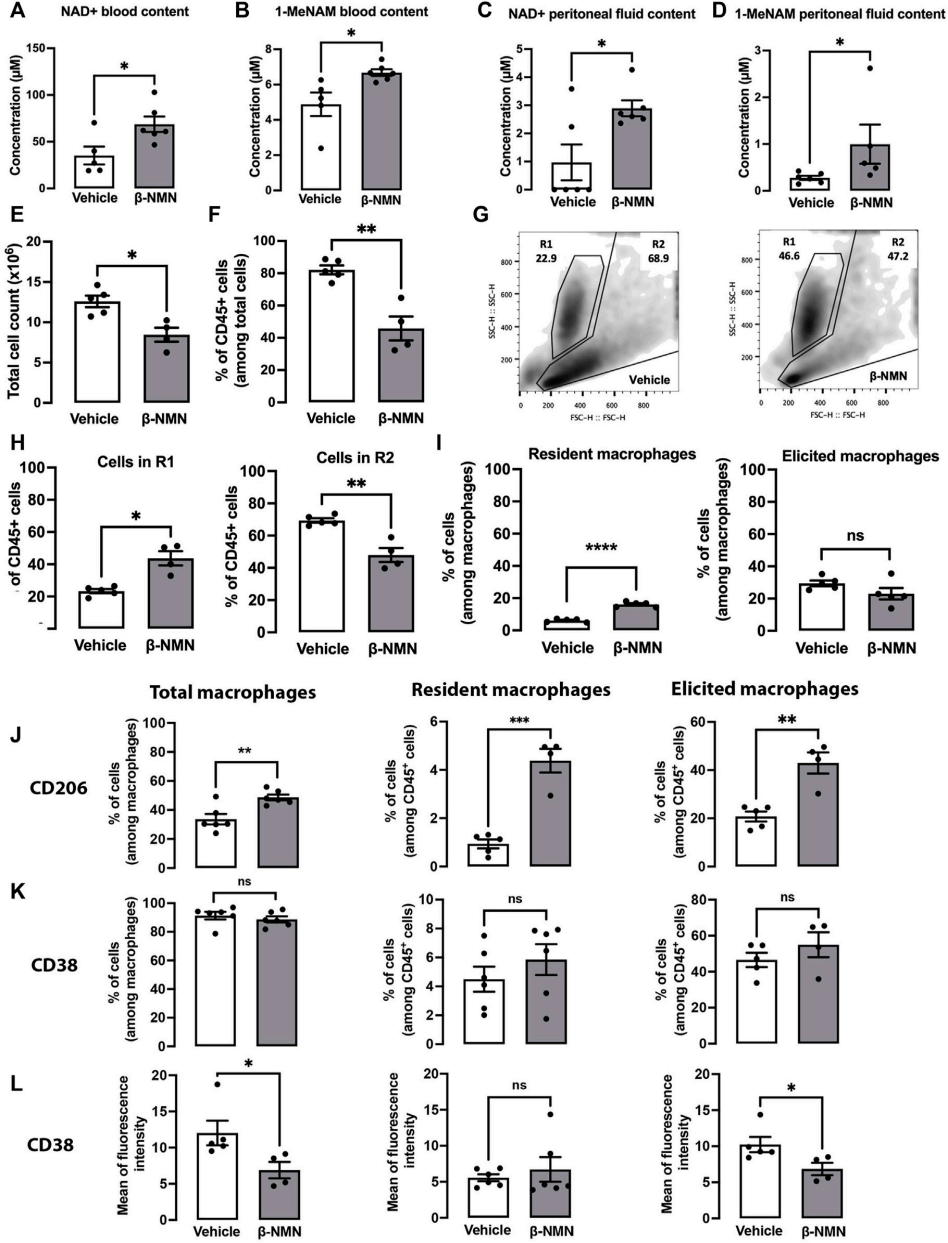
Phenotypic characterization of peritoneal cells from mice subjected to TGC-induced peritonitis treated or not with β-NMN 3 days post TGC administration, blood, and peritoneal exudates from vehicle and β-NMN mice were collected. NAD+ and 1-MeNAM were dosed by UHPLC, and the peritoneal cells were characterized by flow cytometry. **(A–D)** NAD+ and 1-MeNAM contents of blood **(A,B)** and peritoneal fluid **(C,D)** of mice. **(E)** Total cell count in the peritoneal extrudates of mice. **(F)** Proportion of CD45 ^+^ cells found in the peritoneal exudates. Data were expressed as a percentage of total cells. **(G)** Representative dot-blot of forward scatter (FS) versus side scatter (SS) of peritoneal cell populations (R1 and R2) from vehicle- (left panel) and β-NMN-treated (right panel) mice. **(H)** Proportion of total peritoneal cell population in the R1 and R2 gates. Data were expressed as % of CD45 ^+^ cells. **(I)** Proportion of resident and elicited macrophages present in the CD45 ^+^ cell population. Resident and elicited macrophages were discriminated based on: F4/80^+^CD16/32^+^CD64^lo^LFA1^lo^MHC-II^lo^; F4/80^+^CD16/32^+^CD64^hi^LFA1^hi^MHC-II^hi^, respectively. The proportion of different subsets of macrophages was expressed as % of cells among total macrophages. **(J–K)** Proportion of macrophages and subpopulations of macrophages expressing CD206 **(J)** and CD38 **(K)** expressed as % of total macrophages and different subpopulations among the CD45 ^+^ cells. **(L)** Level of CD38 expression on macrophages and subpopulation of macrophages. Data were expressed as the mean of fluorescence intensity. Data are mean ± SEM; n = 4–6 for each group; ns = non-significant; *p < 0.05; **p < 0.01; ***p < 0.001; ****p < 0.0001 vs. vehicle.

We hypothesized that β-NMN may prevent the development and progression of peritonitis that triggers septic shock and multi-organ damage (Barrera et al., 2011). Thus, we evaluated the effect of β-NMN administration on TGC-elicited peritonitis which represents a suitable model to study inflammatory events characterized by inflammatory mediator production and leukocyte accumulation. Mice were injected once with TGC followed by daily injections of either β-NMN or vehicle for the next 3 days, after which blood was collected and peritoneal lavages were performed. Remarkably, β-NMN was able to significantly increase NAD+ and 1-MeNAM levels in blood and supernatants of peritoneal exudates as compared to vehicle administration (Figures 3A–D).

Cell count from the peritoneal exudates showed that the level of the inflammatory response in vehicle-treated animals was in a range comparable to those already reported for mice 4 days after single priming TGC injection as previously reported (Cook et al., 2003).

Interestingly, treatment with β-NMN reduced the number of cells present in the peritoneum by approximately one-third compared to vehicle-treated animals (Figure 3E). Accordingly, the percentage of CD45^+^ cells was significantly lower in peritoneal exudates of β-NMN-treated mice as compared to vehicle-treated animals (Figure 3F), arguing in favor of possible reduced infiltration of immune cells upon β-NMN administration. Flow cytometric analysis of forward scatter (FS) and side scatter (SS) parameters, measuring size and granularity, respectively, revealed morphological and quantitative differences between cells obtained from peritoneal lavages of β-NMN animals compared to vehicle animals (Figure 3G). Among CD45^+^ cells, two populations can be clearly distinguished based on their FS/SS parameters. The first population of homogeneous size and high granularity/complexity is labeled R1 and a second, R2, of variable size and heterogeneous granularity/complexity (Figure 3G). Administration of β-NMN led to a significant increase in R1 and a concomitant reduction in the R2 population (Figure 3H). Furthermore, in the presence of β-NMN, cells in R2 had lower size and granularity/complexity as compared to cells in R2 from vehicle-treated mice (Figure 3G). R2 population was further characterized by the surface markers F4/80, CD64, CD16/32, LFA-1, and MHC-II, to identify the monocyte/macrophage populations. Among those, the proportion of F4/80^+^CD16/32^+^CD64^hi^LFA1^hi^MHC-II^hi^ cells corresponding to elicited macrophages in β-NMN, and vehicle groups remained unchanged, although a tendency to a decrease is observed upon treatment with β-NMN. Conversely, the percentage of F4/80^+^CD16/32^+^CD64^lo^LFA1^lo^MHC-II^lo^, representing resident macrophages, was significantly higher in a β-NMN group compared to vehicle group (Figure 3I).

Interestingly, in comparison to the vehicle, β-NMN treatment significantly increased the percentage of peritoneal macrophages expressing CD206 (C-type mannose receptor 1, MRC1), a marker of mouse and human M2 macrophages (Murray et al., 2014; Porcheray et al., 2005). This was also observed in both sub-populations of residents and elicited macrophages (Figure 3J). On the other hand, β-NMN did not affect the proportions of macrophages expressing the NAD+-consuming enzyme, CD38^+^, a robust marker of M1 macrophage (Jablonski et al., 2015) (Figure 3K) but significantly reduced its surface expression, specifically on the elicited macrophages but not on the resident population (Figure 3L).

### 3.5 β-NMN Treatment During Peritonitis Reduces Phagocytosis Ability, Increases Viability, and Prevents Pro-Inflammatory Secretion in Response to Bacterial Stimulation

**FIGURE 4:**
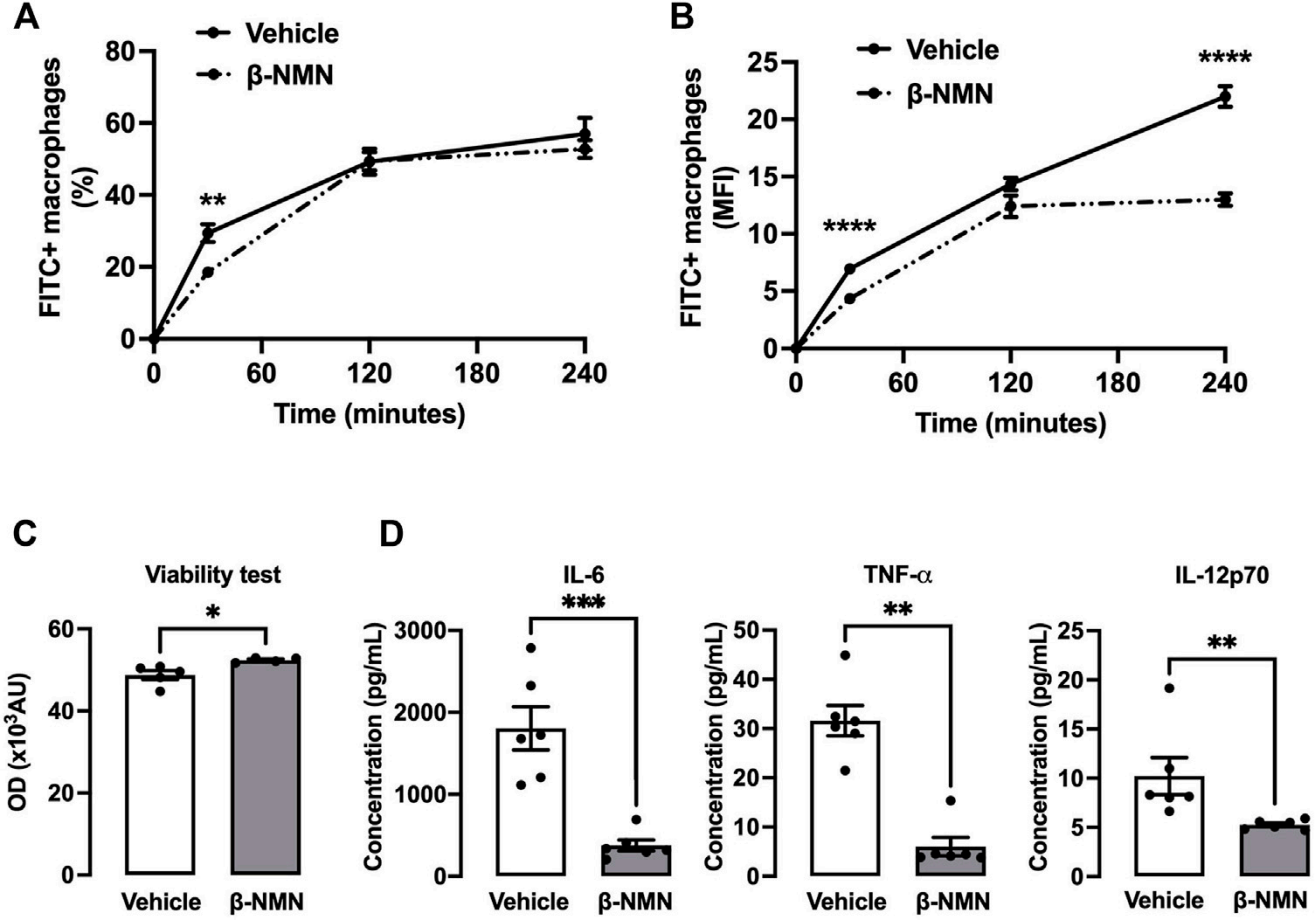
Functional properties of peritoneal macrophages following stimulation with *E. coli* in the TGC-induced peritonitis mouse model. **(A,B)** Phagocytosis of peritoneal macrophages. Three days post TGC administration, macrophages from peritoneal lavage fluid were cultured on plates and stimulated with pH-Rhodo-labeled *E. coli*. The proportion of phagocytic macrophages at 30, 120, and 240 min was analyzed by flow cytometry. The quantity of engulfed *E. coli* was expressed as % of total macrophages **(A)** or as the mean of fluorescence intensity (MFI) **(B)**. **(C,D)** Viability and pro-inflammatory secretion of peritoneal macrophages. Three days post TGC administration, macrophages were co-cultured with *E. coli* for 24 h. **(C)** Viability of macrophages was assessed by the AlamarBlue^®^ assay and expressed as optical density (OD). **(D)** Secreted cytokine concentrations (IL-6, TNFα, and IL-12p70) were determined in the cell culture supernatant by flow cytometry using a bead-based immunoassay. IL = interleukin; TNF-α = tumor necrosis factor-alpha. All data are mean ± SEM; n = 4–6 for each group; *p < 0.05; **p < 0.01; ***p < 0.001; ****p < 0.0001 vs. vehicle.

To assess whether the observed phenotype differences in peritoneal macrophages induced by β-NMN treatment after TGC induction might have functional consequences, we studied their phagocytosis potential using pH-Rhodo-labeled *E. coli* bacteria and their viability and cytokine secretion capacity after bacterial stimulation*.* To evaluate the “phagocytic ability” between macrophages treated with β-NMN and vehicle, we have measured the percentage of cells that were pHrodo-Red positive and the mean fluorescence intensity (MFI). As compared to vehicle, β-NMN treatment did not markedly modify the kinetics of appearance and the proportion of pHrodo-Red positive macrophages. Only a slight reduction was noted after 30 min in presence of bacteria (Figure 4A). Interestingly, macrophages obtained from β-NMN-treated mice had significantly lower MFI after 30 min and 4 h of incubation with pH-Rhodo labeled *E. coli* (Figure 4B), suggesting a reduced number of phagocytosed bacteria per cell.

Additionally, the AlamarBlue^®^ assay revealed that macrophages from β-NMN-treated mice stimulated with *E. coli* for 24 h showed a slight but significant increase in viability (Figure 4C).

Lastly, macrophages from β-NMN-treated animals released 5 times less IL-6, TNF-α and 2 times fewer IL-12p70 compared to macrophages from animals treated with vehicle when exposed to *E. coli* for 24 h (Figure 4D).

### 3.6 RNA-Seq Study of Macrophages Subjected to Bacterial Stimulation Highlights the Dynamics of Transcriptional Changes Induced by β-NMN Treatment

Following TGC-induced-peritonitis, RNA-seq analysis was performed on harvested macrophages stimulated *in vitro* with *E. coli* bacteria for 6 h. Gene expression signature associated with the phenotype and functional differences observed upon β-NMN treatment was then characterized.

**FIGURE 5:**
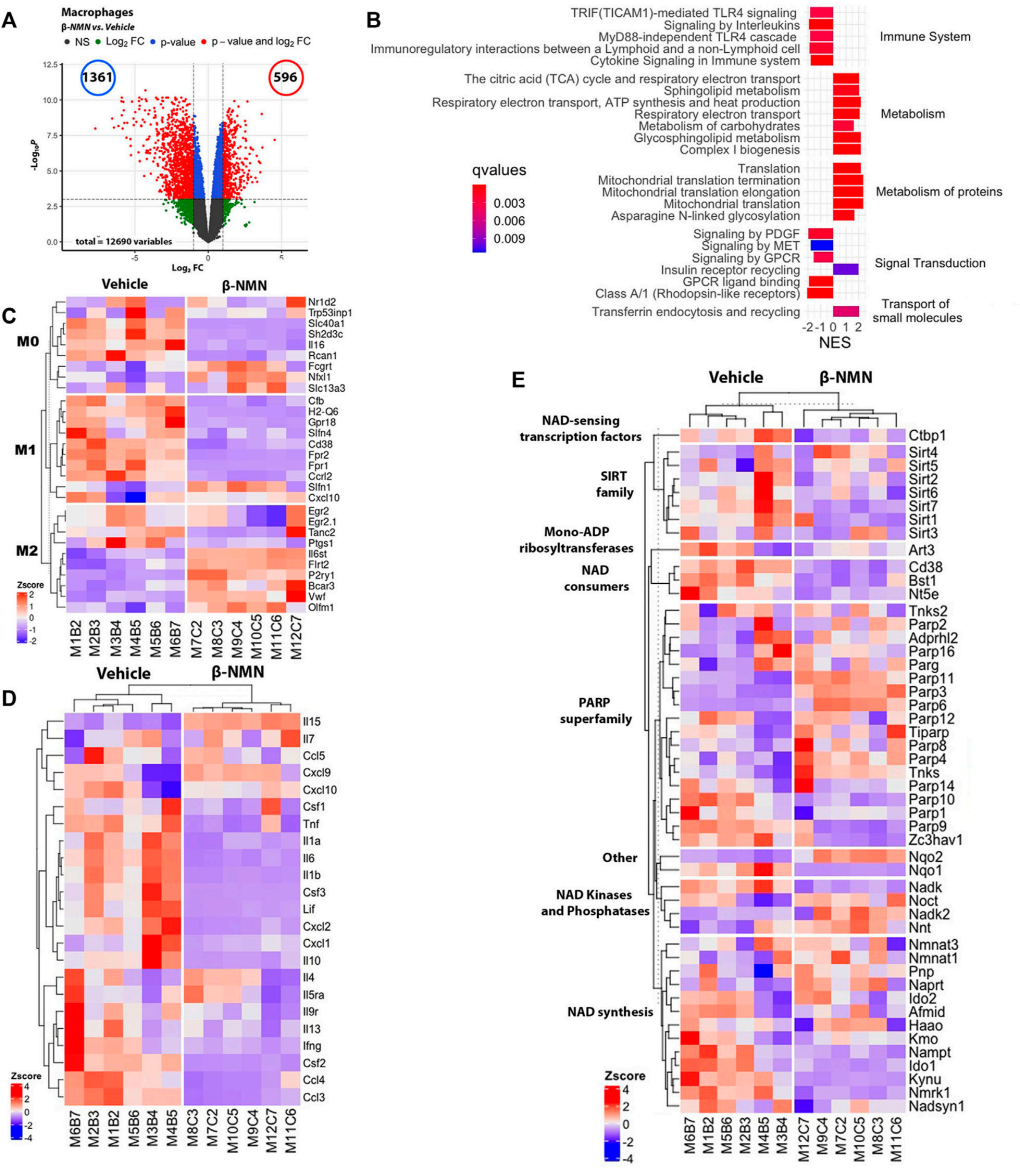
Identification of differentially expressed genes in peritoneal macrophages following stimulation with *E. coli* in the TGC-induced peritonitis mouse model. Peritoneal macrophages were cultured in the presence of *E. coli* for 6 h and then submitted to transcriptomic analysis using *de novo* RNA sequencing. **(A)** Volcano plot of gene expression of peritoneal macrophages [plot of gene expression log2-transformed fold-changes (*x*-axis) vs. -log10 *p*-value (*y*-axis)]. The red dots represent the gene having fold-changes>2.0, and adjusted *p*-values<0.05 between β-NMN and vehicle groups. **(B)** Enrichment plot of differentially expressed Reactome pathways (β-NMN vs. vehicle) in peritoneal macrophages. Normalized enrichment score (NES) reflects the up- (NES>0) and down- (NES<0)expressed pathways, and gradient color reflects the adjusted *p*-values <0.01. **(C–E)** Hierarchical clustering heatmap of M0, M1, and M2 phenotype- **(C)**, cytokine production- **(D)**, and NAD + metabolism- **(E)** associated genes expressed by peritoneal macrophages. Red colors indicate Z-score>0 (up-regulation); blue colors indicate Z-score<0 (down-regulation); white color indicates Z-score = 0 (no difference). For all data *n* = 6 for each group.

Overall, a general heatmap of the obtained RNA-seq data revealed that macrophage samples clustered according to β-NMN or vehicle treatment (Supplementary Figure S3). Differential expression analysis between β-NMN- and vehicle-treated mice identified 7,533 differentially expressed genes with 3,801 being overexpressed and 3,732 downregulated in macrophages from the β-NMN group in comparison to vehicle (Figure 5A).

GSEA analyses using the Reactome database (adjusted *p*-value < 0.05 and abs (NES) > 1) revealed that among the top 24 impacted pathways, 14 and 10 up- and down-regulated pathways, respectively (Figure 5B and Supplementary Table S1). One important feature of the pathways impacted by the treatment is the decreased expression of genes coding for the Toll-Like Receptor (TLR) 4, and its association with downstream cell signaling pathways. Along with this effect on the microbial recognition molecules, peritoneal macrophages from mice administrated with β-NMN showed a reduced expression of the pathways of cytokine signaling, and of antigen presenting through the MHCI class receptor confirming the immunomodulatory effect of the β-NMN treatment during TGC-induced peritonitis on macrophages subjected to bacterial stimulation (Figure 5B).

As observed in the hearts and lungs of mice subjected to CLP, β-NMN administration upregulated the expression of genes involved in the production of cellular energy (i.e., carbohydrate metabolism and insulin receptor recycling) and mitochondrial integrity and functions (i.e., TCA cycle, respiratory electron transport, complex I biogenesis, and mitochondrial translation) (Figure 5B).

We then analyzed the expression of genes involved in the phenotypic polarization of macrophages using M0/M1/M2 markers established previously (Jablonski et al., 2015; Viola et al., 2019). Consistent with the results of functional and secretory capacities in macrophages derived from β-NMN-treated mice, this analysis revealed that β-NMN promoted the expression of genes specific to specifically expressed in M2 macrophages and conversely downregulated a significant number of genes related to the M1 phenotype (Figure 5C). Accordingly, the expressions of most of the genes coding for cytokines and chemokines, except for *Il-4*, *-7*, *-15*, and C*xcl*9 and *10* genes, were strongly reduced after β-NMN treatment (Figure 5D), which corroborates the measurements we obtained for cytokines released by macrophages after activation by bacteria.

Finally, NAD+ metabolism was significantly impacted by β-NMN treatment (Figure 5E). We observed that the presence of β-NMN reduces the expression of the genes coding for NAD+ degrading enzymes (*Cd38, Bst1,* and *Nt5e*) along with decreased expression of genes associated with the NAD+ *de novo* synthesis and recycling (salvage) pathways, suggesting that directly or indirectly NMN exerts negative feedback on the NAD+ biosynthetic pathways. On the contrary, nicotinamide mononucleotide adenylyl transferases (NMNATs) genes, that catalyze the condensation of β-NMN with the AMP moiety of ATP to form NAD+, were markedly upregulated. Additionally, genes coding for sirtuins (SIRTs) 1, 2, 3, 6, and 7, and PARPs 1, 9, 10, and 14 were reduced while genes coding for SIRTs 4 and 5 and PARPs 2, 3, 4, 6, 8, 1, 12, and 16 were upregulated, suggesting different roles played by these enzymes in the pathophysiology of peritonitis and sepsis.

## 4 Discussion

In the present study, we investigated the effect of β-NMN administration on CLP-induced bacterial peritonitis and sterile TGC-induced peritonitis in mice.

We show for the first time that administration of β-NMN (the precursor immediately preceding NAD+ in the salvage pathway) shortly after induction of CLP increased survival and had a positive impact on temperature loss, clinical score, bacterial load, and inflammation (Figure 1 **)**.

Consistent with our results, previous works have shown that boosting NAD+ levels by administering NAD+ or other NAD+ biosynthetic precursors (i.e., Niacin, NAM, and NR) alleviates the polymicrobial sepsis in different animal models. Indeed, it has been shown that the treatment with a high dose of NAD+ prior to a lethal dose of *E. coli* or LPS resulted in a marked increase in survival in mice (Iske et al., 2020). Furthermore, administration of high-dose of Niacin to rats subjected to LPS-induced endotoxemia attenuated lung inflammation, reduced histologic lung damages, and improved survival at 72 h post injection (Kwon et al., 2011). Lastly, NAM treatment increased the survival rate of mice with lethal endotoxemia or CLP-induced polymicrobial sepsis (Yuan et al., 2012). More recently, NR administration was shown to improve survival of mice when i. p injected in the fecal-induced peritonitis model of sepsis (Hong et al., 2018). Although this study and ours investigated the effect of the NAD+ precursor 24 h post-CLP-induction, NR was administered at a dose of 300 mg/kg while we used a lower dose of β-NMN at 185 mg/kg. Furthermore, unlike β-NMN, administration of NR after induction of sepsis provided no protection, only pre-treatment with NR conferred significant benefits in septic mice. These results, combined with existing data on the superior properties in terms of activity and side effects profile of β-NMN compared to other molecules impacting NAD+ metabolism (Poddar et al., 2019), suggest a marked advantage of using β-NMN in the therapeutic treatment of bacterial infections.

In the CLP-induced peritonitis/sepsis model, we did not observe any macroscopic tissue damage at 24 h post-surgery in the heart and lungs (Figure 2F), which are the organs most frequently affected during sepsis (Caraballo and Jaimes, 2019). This absence of obvious histological changes is characteristic of early organ failure during sepsis. Indeed, preservation of tissue architecture with minimal apoptosis, necrosis, or cell damage has been reported in septic patients, suggesting more functional rather than structural alterations (Lewis et al., 2016). Accordingly, transcriptomic analysis of these organs reveals a significant impact of β-NMN administration on the expression of genes related to 1) immuno-inflammatory response, 2) bioenergetic metabolism, and 3) mitochondrial structure and function (Figure 2). These results are even more remarkable if we consider that the effects of treatment on the early stages of bacterial sepsis development were obtained after a single administration of β-NMN.

Interestingly, the pathways modulated by β-NMN administration during the early stages of sepsis are those involved in the development of multi-organ failure in both animal models and humans (Jeger et al., 2013; Lewis et al., 2016; Singer et al., 2016; Kraft et al., 2019; Rumienczyk et al., 2021). Thus, β-NMN would counteract the alterations induced by bacterial infection first by reducing the expression of genes coding for inflammatory molecules and the activation of immune cells, and then by optimizing the capacity of cells to produce energy, promoting both glycolysis and lipid oxidation. β-NMN would prevent cells from falling into an ultimate “hibernation” protective state, described by Singer *et al.* that is characterized by hypometabolism in response to a drastic decrease in mitochondrial respiration and ATP production (Singer et al., 2004; Singer, 2007; Carré and Singer, 2008; Singer, 2014). β-NMN would then allow cells to keep performing their functions within organs and to provide defense against infectious agents.

Similarly, we observed that β-NMN treatment in peritoneal macrophages recruited after TGC injection and subjected to bacterial stimulation, led to a transcriptomic signature comparable to that observed in the heart and lungs following CLP (Figure 5), suggesting that the action of β-NMN on gene expression is a general mechanism that is not restricted to the polymicrobial sepsis model alone and would affect not only immune cells but potentially all organ cells.

There is an increasing body of evidence that associates the NAD+ homeostasis with immune responses during infections, suggesting the potential of modulating the intracellular levels of NAD+ to improve the host’s defense against pathogens (Navarro et al., 2021). However, little is known about how the augmentation of NAD+ levels affects the reprogramming of immune cells, and more precisely the metabolism and polarization states of macrophages, during bacterial infection and immune activation.

Our transcriptomic analysis revealed that the expression of genes coding for NAD+ consuming enzymes such as poly (ADP-ribose) polymerases (PARPs) and glycohydrolases (CD38 and CD157) was enhanced in macrophages harvested from mice that developed TGC-induced-peritonitis and stimulated *in vitro* with bacteria. Concomitantly, the expression of genes involved in *de novo* and recycling NAD+ synthetic pathways were also overexpressed (Figure 5E). These results are in line with recent studies which attribute the depletion of NAD+ seen during inflammatory macrophage response to the increased activity of PARPs (primarily PARP-1) and CD38 triggered by ROS-dependent DNA damage (Cameron et al., 2019), and by proinflammatory Toll-like-receptor ligands (Chini et al., 2020; Covarrubias et al., 2021), respectively. Indeed, the overexpression of CD38 has been shown to be a signature of pro-inflammatory M1 macrophage polarization (Jablonski et al., 2015; Covarrubias et al., 2021). The increased expression of nicotinamide phosphoribosyl transferase (NAMPT), the rate-limiting enzyme in the NAD+ salvage pathway, and of indole-2,3-dioxygenase 1 (IDO1), which catalyzes the first step of the kynurenine pathway observed in macrophages from TGC-treated mice, is likely needed for sustaining the NAD+ content necessary to drive glycolysis and to support the production of pro-inflammatory mediators, as previously reported (Cameron et al., 2019; Minhas et al., 2019; Covarrubias et al., 2021).

Remarkably, β-NMN treatment, while significantly increasing the NAD+ and 1-MeNAM contents in peritoneal fluid (Figure 3C and 3D), completely reverted the overexpression of NAD+ consuming enzymes (CD38, CD156, and PARP1,9,10,14) and of genes involved in *de novo* (Ido1 and Kynu) and recycling (Nampt and NmrK1) NAD+ synthetic pathways. On the contrary, Nmnat 1 and 3, enzymes catalyzing the direct conversion of β-NMN into NAD+, were overexpressed (Figure 5E).

These results suggest that β-NMN treatment was able to counteract the depletion of the NAD+ pool and therefore eliminate the feedback regulation that enhances NAD+ biosynthesis via NAMPT and IDO1. This is particularly relevant since the increase of kynurenine generated by IDO 1 has been associated with loss of microvascular reactivity and immune dysregulation in sepsis (Zeden et al., 2010; Changsirivathanathamrong et al., 2011; Darcy et al., 2011), and the use of nicotinamide to generate NAD+ via NAMPT is limited by its inhibitory function on Sirtuins (Bitterman et al., 2002) and side effects (Bogan and Brenner, 2008).

Lastly, the overexpression of two known oxidative stress-induced genes, Nadk and Nqo1, was reverted upon β-NMN administration (Ross and Siegel, 2017; Tedeschi et al., 2016). These results together with the down-regulation of Parp genes indicate that β-NMN treatment might attenuate the ROS-induced DNA damage during immune activation. One interesting outcome from our data is that β-NMN supplementation appears to impact macrophage reprogramming from a pro-inflammatory M1 phenotype toward an anti-inflammatory/pro resolving M2 profile as demonstrated by the decrease of several inflammatory mediators, including TNF-α, IL-6, and IL-12 (Figure 4D) as well as markers of M1 signature and the concomitant increase of M2 markers (Figure 5C). Furthermore, the enrichment of metabolic pathways involved in the citric acid cycle and respiratory electron transport after β-NMN treatment (Figure 5B) corroborates the shift toward M2 macrophages that are known to rely on oxidative metabolism compared to M1 macrophages that are predominantly glycolytic (Galván-Peña and O'Neill, 2014).

In summary, we demonstrate here that β-NMN improved survival and prevented clinical deterioration in septic mice. β-NMN-treated mice are characterized by a transcriptomic signature that reveals an attenuation of uncontrolled inflammation as well as a sustained immune response sufficient to provide an effective defense against infectious agents. In addition, the observed changes in gene expressions suggest that β-NMN may enhance the ability of cells to produce sufficient energy (through both glycolysis and lipid oxidation) to sustain their functions during bacterial infection.

Thus, in light of our beneficial experimental data and the positive toxicology and safety profiles (Cros et al., 2021), we believe that β-NMN represents a promising therapeutic approach to treat bacterial infections and their deleterious consequences on organs.

## Data Availability

The original contributions presented in the study are publicly available. This data can be found here: https://www.ncbi.nlm.nih.gov/geo/query/acc.cgi?acc=GSE199156.
