# Supplementary figures and images for: Nicotinamide Mononucleotide Administration Triggers Macrophages Reprogramming and Alleviates Inflammation During Sepsis Induced by Experimental Peritonitis
Source: Front Mol Biosci. 2022 Jun 27;9:895028. doi: 10.3389/fmolb.2022.895028 (PMC9271973; doi:10.3389/fmolb.2022.895028)

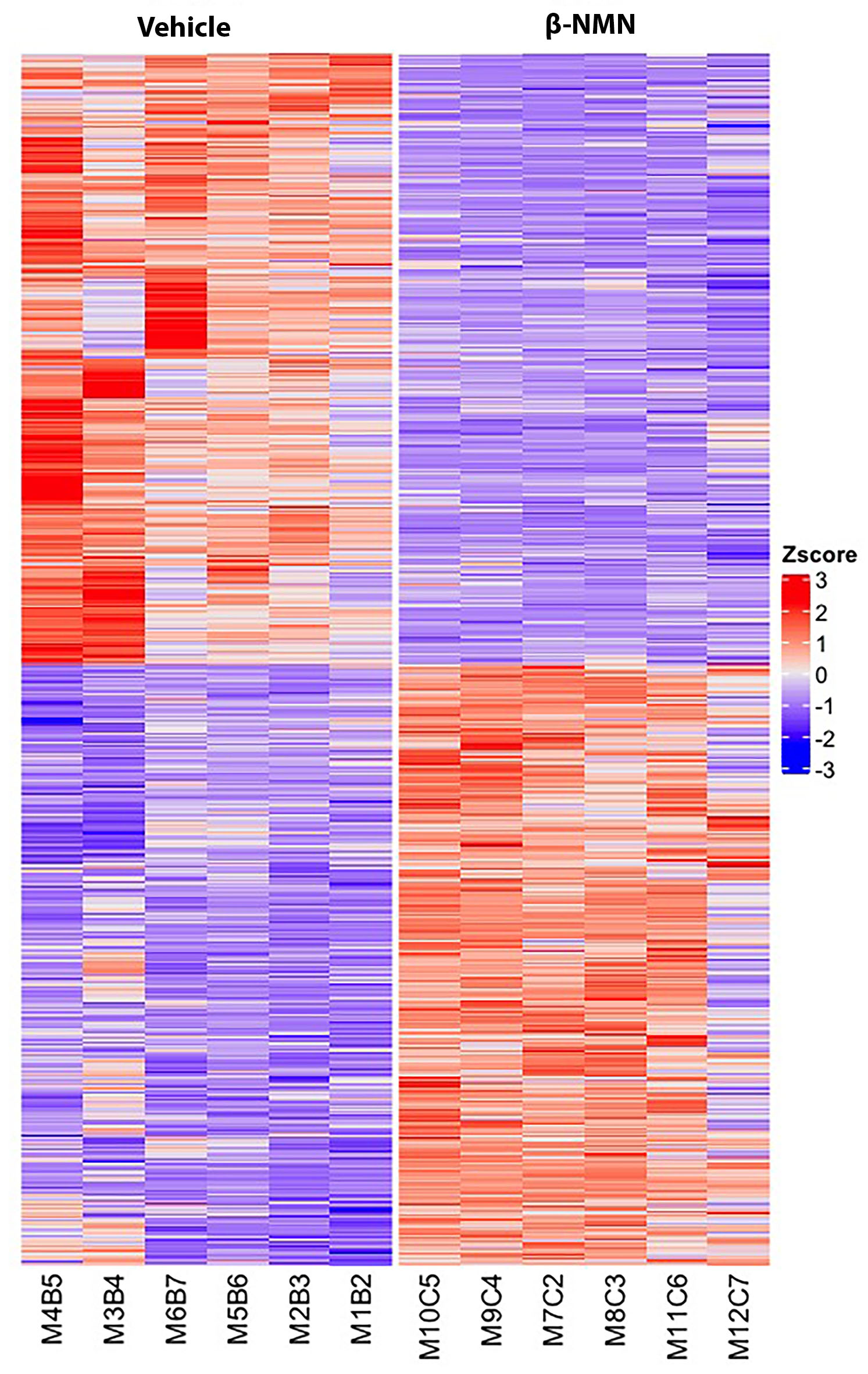

Supplement: Supplementary file 1 [file Image3.JPEG]

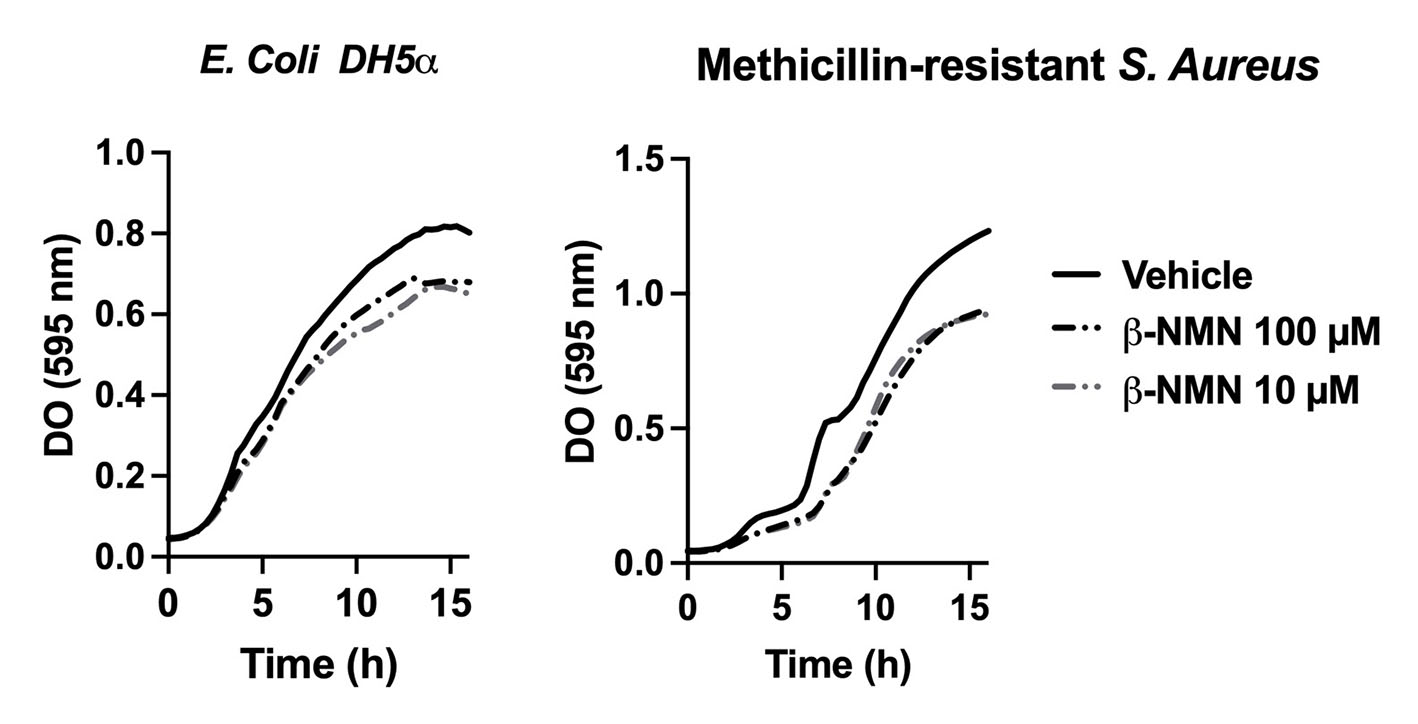

Supplement: Supplementary file 2 [file Image1.JPEG]

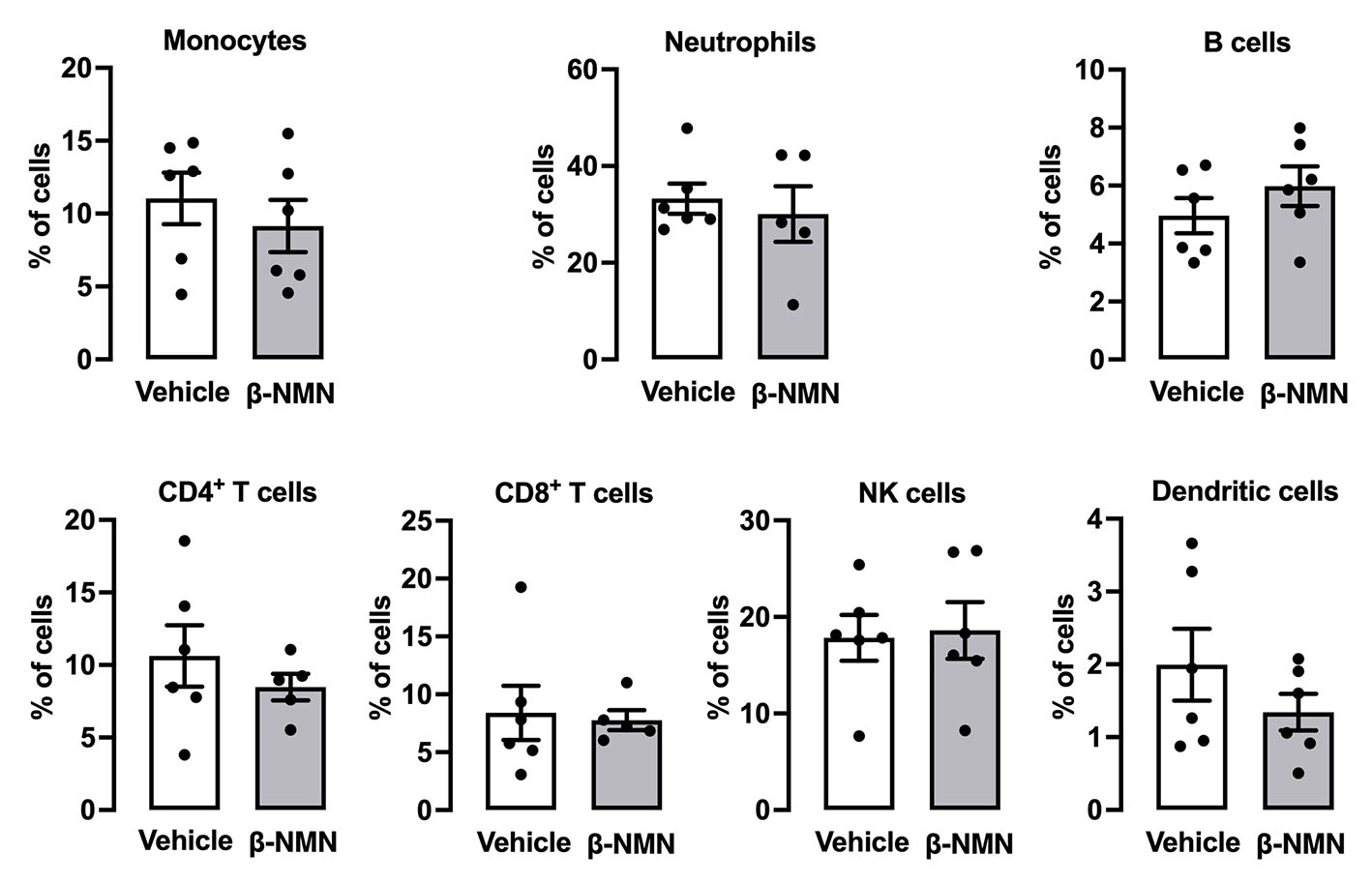

Supplement: Supplementary file 3 [file Image2.JPEG]
