# Supplementary material for: Nicotinamide Mononucleotide Administration Triggers Macrophages Reprogramming and Alleviates Inflammation During Sepsis Induced by Experimental Peritonitis
Source: Front Mol Biosci. 2022 Jun 27;9:895028. doi: 10.3389/fmolb.2022.895028 (PMC9271973; doi:10.3389/fmolb.2022.895028)
Supplement: Supplementary file 5 [file DataSheet1.docx]

Supplementary Material

# Supplementary Materials and Methods

## Blood cell immuno-phenotyping

Blood immune cell characterization derived from mice in cohort 1 of CLP model was carried out by flow cytometry using direct staining. To identify macrophages, monocytes, dendritic cells and neutrophils, blood cells were labeled with following antibodies: CD11b-APC, CD38-FITC, Ly6C-PeVio770, CD11c-Vioblue, Iab-V500, Ly6G-PE (Miltenyi Biotec, Bergisch Gladbach, Germany). To identify, B cells, T cells and NK cells, blood cells were labelled with following antibodies: TCRβ-APC, CD4-Viogreen, CD8-VioBright FITC, CD19-Vioblue, NK1.1-PeVio770, CD38-PE (Miltenyi Biotec, Bergisch Gladbach, Germany). Briefly, after red blood cells lysis, leukocytes were resuspended in FACS buffer (Phosphate-buffered saline supplemented with 0.5% bovine serum albumin and 0.1% NaN3) prior to incubation with the antibodies. After 20 minutes incubation at 4°C, samples were washed with FACS buffer and cells were characterized on a MACSQuant VYB flow cytometer and analyzed using FlowLogic software.

## Bacterial growth

Methicillin-resistant *S. aureus* 198 and *Escherichia coli* DH5α isolates were a kind gift from Dr Cédric Jacqueline (CR2TI, Team 6 "impact of acute inflammation on host-pathogen interaction and lung homeostasis", Nantes, France). Bacteria were grown overnight at 37°C in Brain Heart Infusion (BHI) broth (Becton-Dickinson, Franklin Lakrs, NJ, USA). After centrifugation at 800 g for 10 minutes, the bacterial pellet was washed twice with 0.9% NaCl. The pellet was then resuspended in BHI and the inoculum was calibrated by nephelometry. Methicillin-resistant *S. aureus* 198 and *Escherichia coli* DH5α growth kinetics in presence of vehicle or β-NMN (10 µM or 100 μM) were monitored using a spectrophotometer Multiskan™ FC (Thermo Fisher Scientific, Waltham, MA, USA). The OD600 variations were recorded over a period of 15 hours and the obtained values were compared to the vehicle.

# Supplementary figure legends

**Supplementary Figure 1.** Growth curves of Escherichia coli DH5𝛼 and Methicillin-resistant S. Aureus in presence of β-NMN (10 and 100µM). Escherichia coli DH5𝛼 and Methicillin-resistant S. aureus were grown at 37°C in BHI medium supplemented with vehicle or β-NMN (10 µM or 100 µM). The OD600 variations were recorded over a period of 15 hours and the obtained values were compared to the vehicle.

**Supplementary figure 2.** Blood Immune cell characterization in the CLP-induced septic mouse model. Twenty-four hours post CLP, blood from vehicle and β-NMN mice was collected, and immune cells were identified by flow cytometry analysis. Blood cells were labeled with CD11b, CD38, Ly6C, CD11c, Iab, Ly6G to identify monocytes, dendritic cells, and neutrophils, or with TCR, CD4, CD8, CD19, NK1.1, CD38 to identify B cells, T cells and NK cells. The proportion of immune cells was expressed as the percentage of total blood cells. Data are mean ± SEM; n=5-6 for each group.

**Supplementary figure 3.** General heatmap of differentially expressed genes in peritoneal macrophage following stimulation with *E. coli* in the TGC-induced peritonitis mouse model. Three days post TGC administration, macrophages from peritoneal lavage fluid were cultured on plates and stimulated with *E. coli* for 6 hours. Stimulated macrophages from vehicle and β-NMN mices were then characterized using *de novo* RNA sequencing. Red colors indicate Z-score>0 (up-regulation); blue colors indicate Z-score<0 (down-regulation); white color indicates Z-score=0 (no difference). n=6 for each group.
